# Impact of the COVID-19 pandemic on the Andalusian program for early detection of diabetic retinopathy: a population-based study

**DOI:** 10.3389/fcdhc.2025.1631252

**Published:** 2025-08-14

**Authors:** Reyes Ravé Garcia, Pablo Rodríguez de Vera Gómez, Eduardo Mayoral Sánchez, Manuel Aguilar Diosdado, María Asunción Martínez-Brocca

**Affiliations:** ^1^ Department of Endocrinology and Nutrition, University Hospital Virgen Macarena, Seville, Spain; ^2^ Andalusian Comprehensive Healthcare Plan for Diabetes, Regional Ministry of Health, Andalusian Public Health System, Seville, Spain; ^3^ Endocrinology and Nutrition, Hospital Puerta del Mar, Cadiz, Spain; ^4^ Biomedical Research and Innovation Institute of Cadiz (INiBICA), Cadiz, Spain

**Keywords:** diabetic retinopathy, risk factor, vision-threatening diabetic retinopathy, type 1 diabetes mellitus, COVID-19

## Abstract

**Aims:**

To analyze the impact of the COVID-19 pandemic on the activity and outcomes of the Andalusian Program for Early Detection of Diabetic Retinopathy (APDR).

**Methods:**

A retrospective observational study was conducted during 2018–2023. The following variables were analyzed annually: newly included patients, retinal photographs performed, and pathological findings categorized by severity. Moderate-to-severe non-proliferative and proliferative retinopathy were classified as vision-threatening diabetic retinopathy (VTDR).

**Results:**

In 2020 and 2021, the number of newly included patients (11,897 and 18,343, respectively) and retinal photographs performed (39,667 and 64,092, respectively) decreased compared to previous years (2018 and 2019: 25,940 and 30,807 new patients, respectively; 85,171 and 100,849 retinal photographs, respectively). In 2022, activity levels increased 160% compared to 2019. The proportion of VTDR cases remained stable at 0.163%, 0.14%, and 0.075% during 2021, 2022, and 2023, respectively, compared to the prepandemic period (0.168% and 0.117% in 2018 and 2019, respectively).

**Conclusions:**

Despite the slowdown in activity during the COVID-19 pandemic, the post-pandemic proportion of VTDR cases diagnosed through the APDR remained stable, suggesting resilience against severe outcomes despite healthcare disruption. The program has demonstrated an effective recovery. Ongoing long-term monitoring is essential to fully assess the pandemic’s potential impact on late-stage complications.

## Introduction

Diabetes mellitus (DM) is a chronic disease that affects 537 million adults worldwide, and this number is expected to rise to 783 million people with DM by the year 2045 (1). In Spain, the prevalence is 13.6% of the adult population, with type 2 diabetes mellitus (T2DM) being the most prevalent form. The incidence is estimated at 11.6 cases of DM per 1000 persons/year, with projections indicating that by 2030, one in ten adults in the country will be affected by this condition (2, 3). It is estimated that over one-third of adults with DM are undiagnosed (4).

Diabetic retinopathy (DR) is a common microvascular complication of DM, which is typically classified into two stages: non-proliferative DR (early stage) and proliferative DR (advanced stage). It is described as a neurovascular impairment of the retina in individuals with either type 1 diabetes mellitus (T1DM) or T2DM (5, 6). DR is considered the leading cause of blindness in adults of working age, and up to 80% of cases are preventable through early detection, timely treatment, and comprehensive management of DM (7–9).

Several studies have assessed the prevalence of DR at both global and regional levels. Thomas et al. (2015–2019) reported a global prevalence of 27%, with the lowest rates observed in Europe (20.6%) and Southeast Asia (12.5%), and the highest in developing regions (10). Li et al. found a DR prevalence of 26.5% in Southern European countries (Portugal, Spain, and Italy), comparable to 25.7% in the rest of Europe. Among individuals with T2DM, the annual incidence of DR was 4.6**%** (11). Additionally, a meta-analysis by Romero-Aroca et al. showed a decline in DR prevalence in Spain, from 28.85% during 2001–2008 to 15.28% between 2009 and 2020, indicating a favorable trend over time. The authors attributed these trends to improved diabetes diagnosis and better metabolic control, noting that DR epidemiology in Spain is influenced by independently managed regional screening programs, leading to a lack of unified national data (12).

Although the prevalence of all stages of this microvascular complication has declined since 1980 in populations with better diabetes control, global studies report an increase in visual impairment and blindness due to DR between 1990 and 2015, mainly driven by the growing diabetes burden, especially in low- and middle-income countries (13). The duration of DM, treatment type, and metabolic control remain key risk factors. Despite advances in screening, glycemic management, and treatment, DR prevalence continues to rise, largely due to the longer life expectancy of people with diabetes.

All of this highlights the importance of preventive efforts targeting the early stages of DR to prevent progression to more advanced stages, thereby enabling early diagnosis and treatment (14, 15).

The region of Andalusia, which covers an area of 87,597 km² in southern Spain, has a population of 8.4 million (18% of the country’s total population). The prevalence of DM is higher (15.3%) than in the rest of Spain (12.5%), closely related to lifestyle and socioeconomic factors (16).Thus, Andalusia, like the rest of Europe, is facing a rapid increase in its population living with chronic diseases (specifically DM), which places significant pressure on its public healthcare systems.

The Public Health System of Andalusia (SSPA) provides universal healthcare through a broad network of 1,500 primary care centers and 49 hospitals, with a focus on accessibility, quality, and patient-centered care, funded by public resources. Screening is a cost-effective strategy to prevent vision loss and is commonly performed with non-mydriatic retinal photography, with annual or biennial intervals recommended by scientific bodies (17–20). Based on this evidence, the Andalusian Program for Early Detection of Diabetic Retinopathy (APDR) was launched in 2005 as part of the Andalusian Comprehensive Diabetes Plan (PIDMA), targeting systematic screening of the diabetic population at risk for DR. Fifteen years of results confirm that the program effectively meets screening needs, optimizes resources, detects asymptomatic disease, and reduces visual impairment (21).

The coronavirus disease (COVID-19), caused by the SARS-CoV-2 infection, and the resulting pandemic that emerged in 2020, led to a profound healthcare and social crisis. During the COVID-19 pandemic, lockdown measures—though essential to control infection and reduce mortality—led to delays in non-COVID-related care due to mobility restrictions, fear of contagion, and isolation recommendations. This disrupted follow-up and treatment of chronic conditions, including diabetes, and hindered the continuity of care for non-communicable diseases. As a result, the pandemic not only impacted COVID-19-related morbidity and mortality but also compromised chronic disease management, particularly for DM.

This study aims to assess the pandemic’s impact on the Andalusian Program for Early Detection of Diabetic Retinopathy (APDR) by analyzing quality indicators, population coverage, and early detection capacity. To our knowledge, this is the most comprehensive evaluation published on a systematic DR screening program. These findings may guide improvements and preparedness for future disruptions affecting vulnerable populations.

## Materials and methods

### Study design and participants

An observational retrospective study was designed to analyze the performance of the APDR during the pandemic period (2020–2023) compared to the pre-pandemic period (2018–2019).

Patients included in the APDR during the study years 2018–2023 were analyzed. The program targets the entire Andalusian population diagnosed with DM who are eligible for screening to detect DR according to clinical practice guidelines, with no prior known diagnosis of DR.

As an exclusion criterion, patients with previously known DR were not included.

### Data sources and study population

Data were specifically analyzed using the APDR records, available since 2005, for the period 2018–2023. The following indicators were evaluated: the number of patients included in the APDR and the total number of retinal photographs performed.

The APDR is a systematic screening program, and during the pandemic, the following specific modifications were implemented:

- During 2020 and 2021, the inclusion of new patients or their follow-up was not prioritized.
- Starting in the last quarter of 2021, following the end of the second state of emergency on May 9, 2021, a proactive re-engagement of patients with DM was initiated by primary care teams in our region.

The study’s population data represents a dynamic cohort in which individuals may enter (upon diagnosis of DM) or exit (due to confirmed DR, withdrawal from the program, or patient death).

The APDR currently operates as follows: Patients with DM are invited to undergo DR screening according to national clinical practice guidelines. In primary care centers and endocrinology services at hospitals, trained nurses perform the retinal photographs. The results are stored in the patients’ electronic medical records within the corporate system (Diraya). Retinal photographs showing signs of DR are classified according to the International Diabetic Retinopathy Severity Scale (22). The images are categorized as: no DR, mild, moderate, severe non-proliferative DR, or proliferative DR. In cases where classification differed between eyes, the most severe result was used. Patients with a screening result of severe non-proliferative DR or worse were classified as having vision-threatening DR (VTDR). The detection of DR is performed through the evaluation of retinal photographs: by a trained family physician for patients with T2DM and by an endocrinologist for patients with T1DM (first screening level). Patients with negative results are scheduled for the next screening cycle according to clinical practice guidelines (19). Retinal photographs with pathological or inconclusive results are referred to an ophthalmologist (second screening level) for confirmation. Patients with confirmed DR or any other pathological findings are managed through the digital platform and referred to the Ophthalmology Department for examination, diagnostic confirmation, follow-up, and treatment when deemed appropriate. A detailed description of the APDR is provided in the article by Rodríguez-Acuña et al. (21).

### Statistical analysis

Descriptive statistical techniques were used to analyze the variables in the study. Absolute values were considered, such as the number of patients included in the program and the total number of retinal photographs performed. The results of the retinal photographs were classified, and the cumulative incidence for each category was calculated by dividing the number of newly identified cases by the total number of retinal photographs conducted in patients without a prior diagnosis of DR. This approach allowed for the assessment of the number of retinal photographs associated with a significant risk to vision.

All statistical analyses were performed using RStudio software, version 1.2.1335 (RStudio, Boston, USA).

### Ethics committee

This study complied with the legal requirements of our regional ethics committee with registration number (2024999013510775) in accordance with the revised guidelines of the Declaration of Helsinki.

## Results

A total of 552,302 retinographies were analyzed within the framework of the APDR program during the study period (2018–2023), corresponding to a total of 127,614 screened patients as of December 2023.

**Figure 1:**
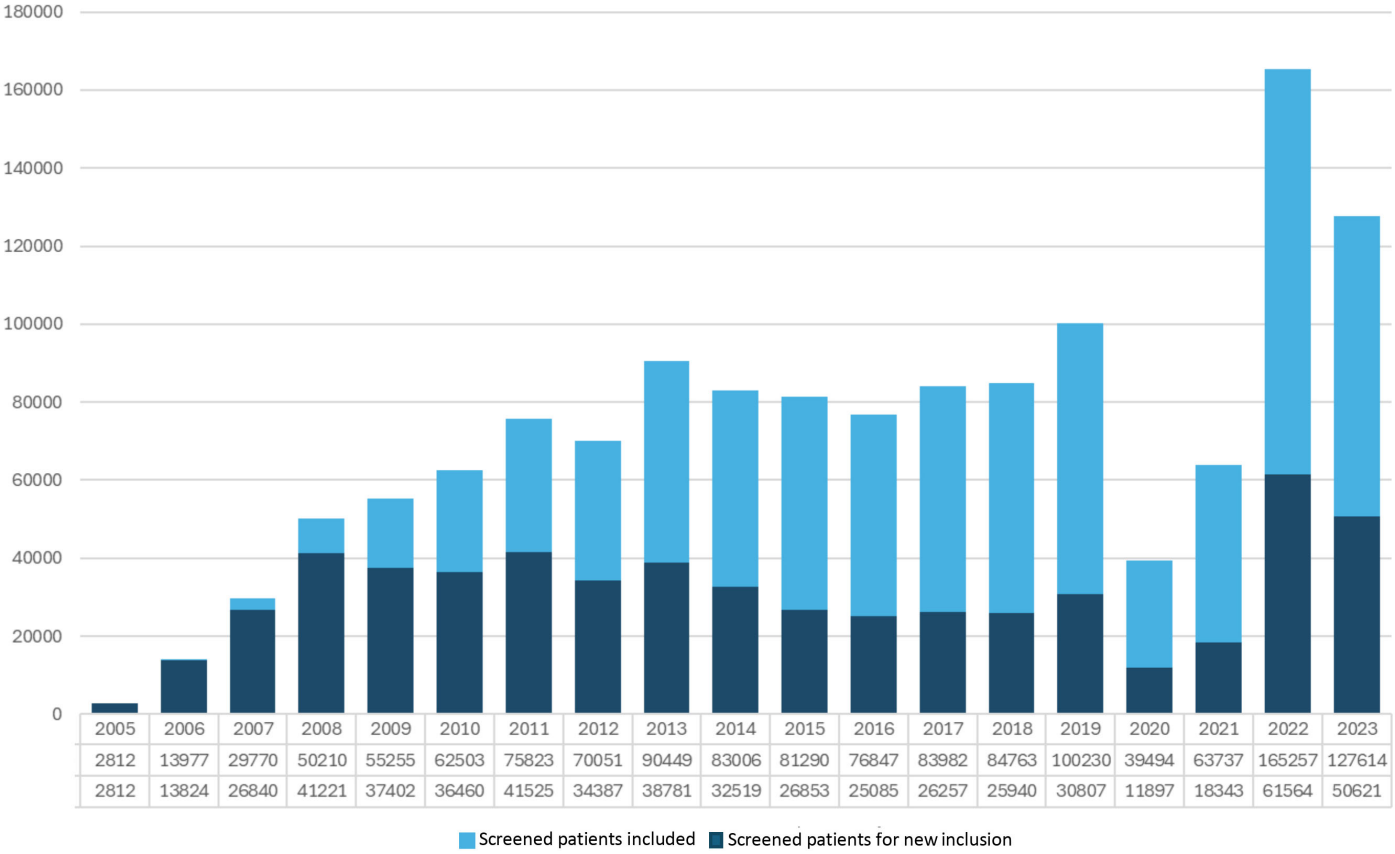
Patients included with completed tests and newly enrolled patients in the Andalusian Program for Early Detection of Diabetic Retinopathy (2005-2023).

We observed that in 2020, the number of screened patients for new inclusion and those with retinal photographs performed during that year decreased compared to previous years. This trend continued throughout 2021. In 2022, there was a 160% increase compared to 2019 in the number of new patients and active participants in the program. During 2023, the number of patients decreased compared to 2022, but the number of new and active patients remained above pre-pandemic levels. **Figure 1** *shows the number of screened patients for new inclusion in the program per year and total screened patients included, from the beginning of the APDR in 2005 to the final year of the study in 2023.*

**Figure 2:**
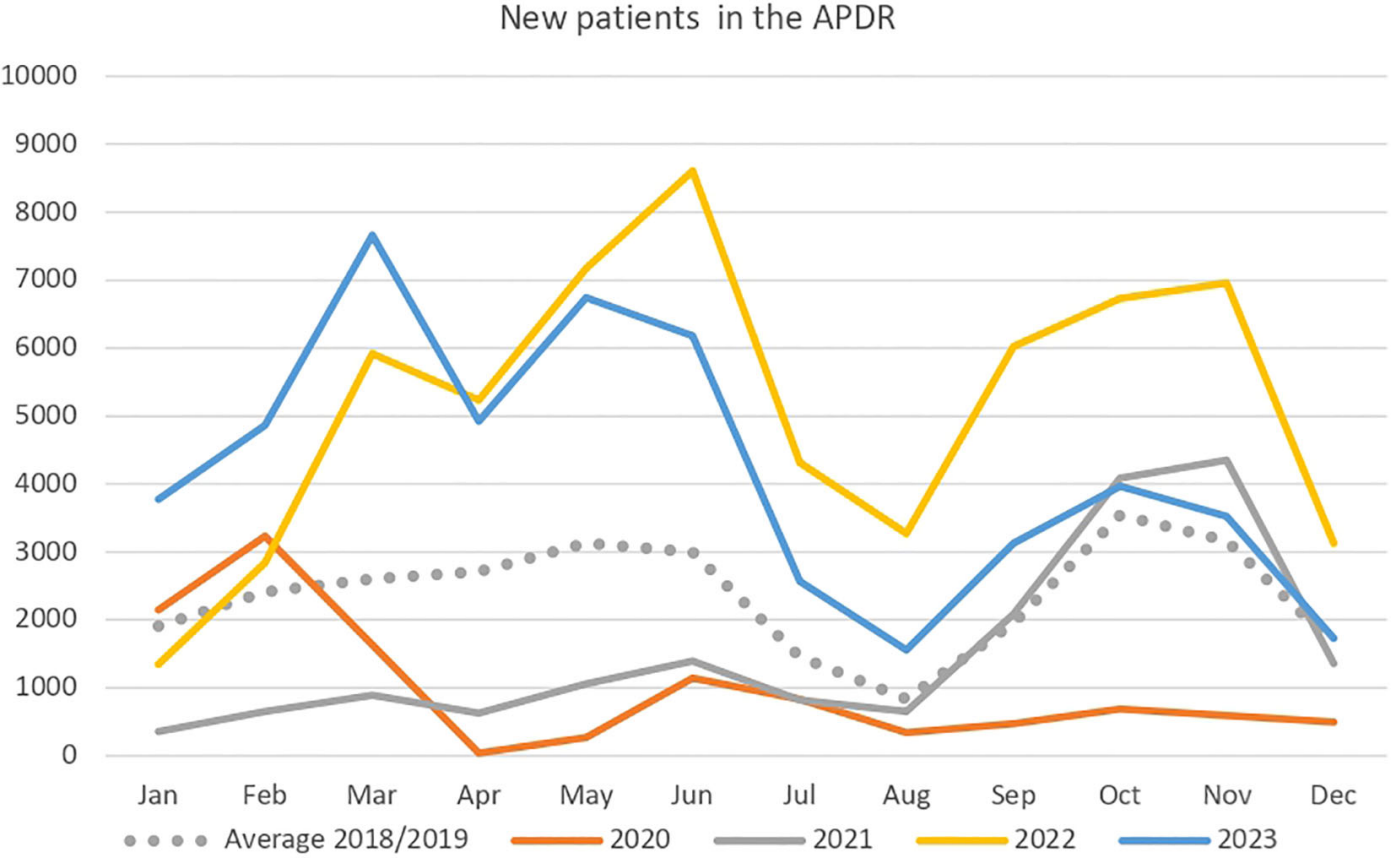
Number of newly enrolled patients in the Andalusian Program for Early Detection of Diabetic Retinopathy (2018-2023).

During the strict lockdown months from March to June 2020, the program’s operation came to a halt, resulting in a decrease in the number of patients included. In the subsequent months, and nearly until the summer of 2021, the number of patients in the program remained below the average of previous years. Subsequently, a recovery trend was observed in 2022, with the number of patients included surpassing the pre-pandemic average, continuing this upward trend until the second half of 2023, when it aligned with the averages recorded prior to the onset of COVID-19 in 2018–2019. **Figure 2** presents a comparative analysis of newly included patients in the APDR, disaggregated by month over the study period, to evaluate the modifications experienced by the program due to seasonal variations.

**Figure 3:**
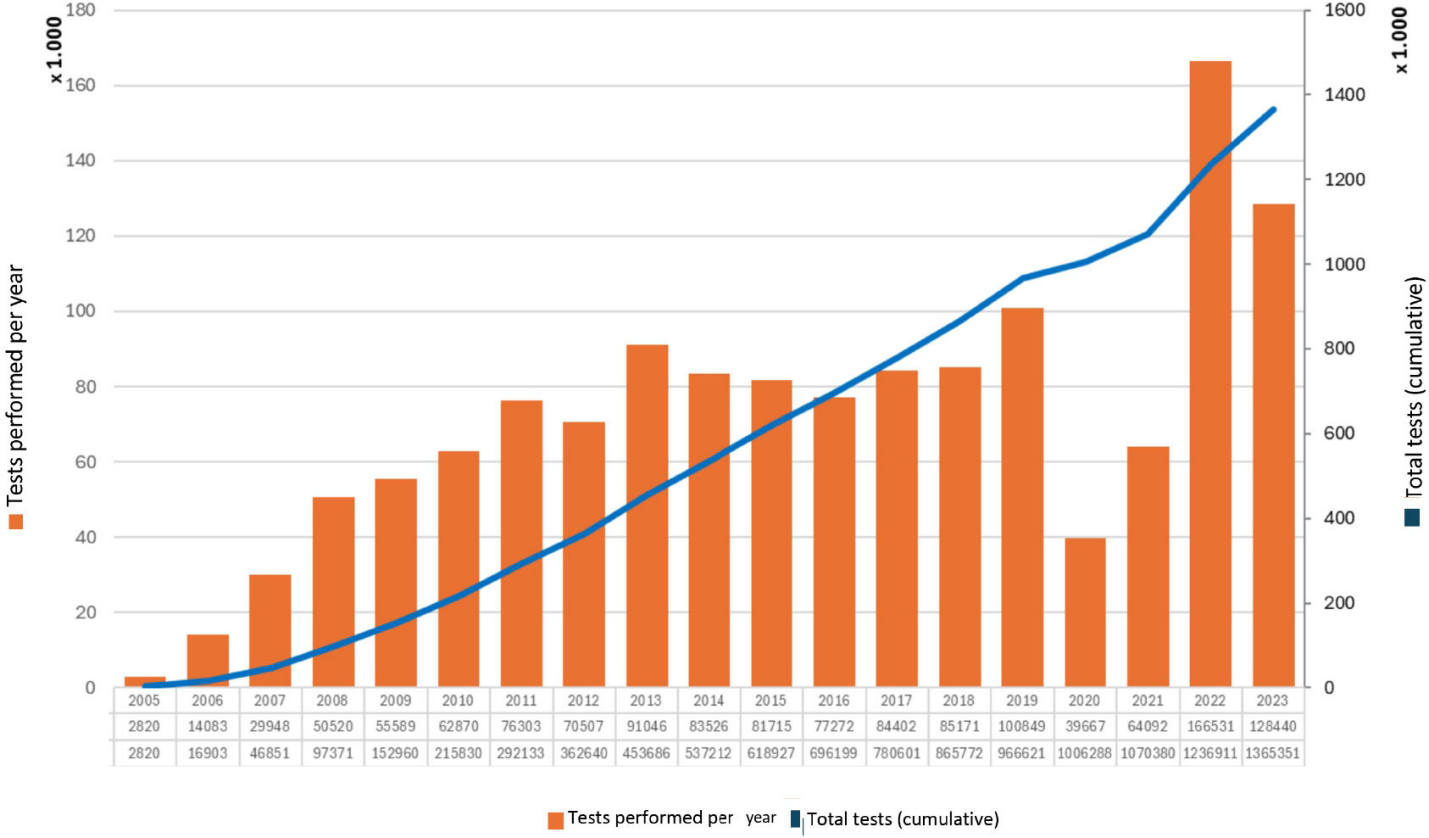
Retinal photographs performed annually in Andalusia within the framework of the Andalusian Program for Early Detection of Diabetic Retinopathy (2005-2023).

**Figure 3** shows the number of retinal photographs performed within the framework of the APDR from its inception in 2005 to the study’s conclusion in December 2023. The annual number of tests has progressively increased since the program’s launch, and the cumulative total of retinal photographs is also represented. However, following the onset of the COVID-19 pandemic in 2020, a drastic reduction in the number of retinal photographs performed was observed, and the program did not recover to pre-pandemic levels in 2021. In 2022, the number of tests performed increased compared to previous years since the APDR’s inception. This upward trend appears to have slowed in 2023 but remains higher than in years preceding the pandemic.

The total number of tests performed classified as VTDR during the study years 2020, 2021, 2022, and 2023 and the five preceding years was evaluated. Results of moderate-to-severe non-proliferative diabetic retinopathy (NPDR) and proliferative diabetic retinopathy (PDR) screened and confirmed are presented, and the percentages for each category are shown in **Table 1**. It was observed that, although the percentage of retinal screenings significantly decreased in 2020, the proportion of VTDR cases remained comparable to previous years, as well as to the years that followed after the reactivation of the APDR.

**Table 1:** Classification of Diabetic Retinopathy severity within the framework of the Andalusian Program for Early Detection of Diabetic Retinopathy (2015-2023).

Years	NO DR	Mild NPDR	Severe moderate NPDR (a)	PDR (b)	VTDR (a+b)	DR Known and treated	Unassessable	Other	Total number of tests
2015	69711 86,85%	3654 4,55%	130 0,16%	28 0,035%	158 0,195%	465 0,58%	4377 5,45%	1902 2,37%	80267
2016	65601 86,54%	3439 4,54%	122 0,16	36 0,047%	158 0,207%	438 0,57%	4408 5,81%	1756 2,32%	75800
2017	71440 86,38%	3659 4,42%	123 0,15%	44 0,053%	167 0,203%	472 0,57%	5207 6,29%	1761 2,13%	82706
2018	71427 85,96%	3376 4,06%	100 0,12%	40 0,048%	140 0,168%	351 0,42%	5706 6,86%	2085 2,51%	83085
2019	85179 86,37%	4233 4,29%	79 0,08%	37 0,037%	116 0,117%	525 0,53%	5866 5,95%	2700 2,73%	98619
2020	33642 87.2%	1537 3,98%	41 0,11%	11 0,028%	52 0,138%	272 0,70%	1993 5,16%	1067 2,76%	38563
2021	54184 87,2%	2609 4,19%	77 0,12%	27 0,043%	104 0,163%	334 0,54%	3155 5,07%	1751 2,82%	62137
2022	135752 85,75%	6429 4,06%	174 0,11%	49 0,030%	223 0,14%	838 0,53%	9218 5,82%	5842 0,53%	158302
2023	97401 87,28%	3844 3,44%	48 0,043%	36 0,032%	84 0,075%	606 0,54%	6380 5,72%	3281 2,94%	111596

DR, Diabetic Retinopathy; Mild NPDR, Mild non-proliferative diabetic retinopathy. Severe moderate NPDR, moderate diabetic retinopathy; PDR, Proliferative diabetic retinopathy; VTDR, vision-threatening diabetic retinopathy (severe-moderate NPDR + PDR).

## Discussion

This study analyzes how the COVID-19 pandemic affected a systematic diabetic DR screening program in a large region of southern Spain, Andalusia. The results presented include a descriptive analysis of the APDR, examining the number of retinal photographs performed, the number of patients included, coverage rates, and the percentage of vision-threatening retinopathy (moderate-severe non-proliferative and proliferative retinopathy) during the 2020–2023 period, compared to the pre-pandemic period (2018–2019). To our knowledge, this is the largest study analyzing the volume of patients included in a DR screening program.

As previously mentioned, the health emergency created by the onset of the COVID-19 pandemic led to significant interruptions in screening programs and healthcare delivery as it was previously known (23). In 2020, the World Health Organization (WHO) conducted a survey that revealed at an international level that a substantial number of healthcare services were partially or completely disrupted: Hypertension services (53%), Oncology services (42%), Care for patients with DM and its complications (49%), Cardiovascular disease services (31%). Additionally, public screening programs were postponed in 50% of the surveyed countries, such as breast and cervical cancer detection programs (23). On the other hand, various alternatives were developed to address the mobilization of nearly all human and healthcare resources toward managing the pandemic. For instance, telemedicine played a crucial role during the strictest lockdown periods, helping to maintain chronic disease care (23).

The APDR has been active in the region of Andalusia since 2005. The results of its implementation from 2005 to 2019 were recently published by Acuña et al. and The Andalusian Comprehensive Plan for Diabetes. A total of 407,762 individuals with DM who had at least one successful retinal photograph during the study period were included. Of the retinal photographs performed: 84.3% were classified as non-pathological, 5.9% detected DR, with 94.2% classified as mild or moderate, and 1.5% (6,256 patients) were classified as severe or proliferative DR. The authors observed that the annual incidence risk of developing DR per patient progressively decreased from 22.0% to 3.2%. Therefore, they concluded that after 15 years of implementation, the APDR has proven to be feasible in this population, effectively addressing the screening needs, optimizing resources, and facilitating the identification of disease in asymptomatic stages (21).

The stability of the APDR over the last two decades has enabled a robust trend analysis of its recruitment capacity and results. A recent report by the Spanish Ministry of Health highlighted the APDR as the only systematic screening program within Spain’s National Health System (SNS). The program has proven effective in improving population coverage, reducing initial annual screenings among diabetics, lowering DR incidence, and decreasing legal blindness rates compared to opportunistic screening (24). From the start of the strict lockdown in March 2020, the APDR experienced a slowdown in its operations due to the health policies implemented during the pandemic. This was reflected in the number of retinal photographs performed and the number of patients included in the program. This situation persisted until September 2021, when the screening program was reinstated. Upon resumption of the APDR, there was an upward recovery in its performance, with an increase in the number of retinal photographs performed and the number of patients included compared to pre-pandemic years. This indicates that, two years after the pandemic, the APDR has successfully recovered.

To assess whether the screening delay may have negatively impacted the early diagnosis of DR, we analyzed the percentage of retinal photographs classified as vision-threatening DR during the study years. Our data suggest stability in the percentage of VTDR forms (moderate-to-severe non-proliferative DR and proliferative DR), despite a higher number of retinal photographs being performed compared to the years preceding the pandemic.

The delay in diagnosing DR and its impact on health outcomes related to the onset of the pandemic has not been extensively studied. A recent review of 31 articles investigated the impact of natural disasters and pandemics on the care and treatment adherence for DR in Puerto Rico. According to the authors, barriers to healthcare access in such exceptional situations increase significantly, leading to delays in treatment, reduced detection and diagnosis of DR, and subsequently worsened visual outcomes for these patients. The authors emphasized the need for developing effective emergency plans specifically designed for this condition (25) Other studies have assessed changes in therapeutic patterns in global clinical practice regarding DR management. In parallel, a decrease in visual acuity was observed in the long-term follow-up of these patients (26). However, in our study, despite the years during which the APDR operated at a slower pace, the percentage of retinal photographs showing sight-threatening DR (VTDR) did not increase compared to pre-pandemic years. This suggests that the final health outcomes, in terms of visual disability caused by DR, were not negatively impacted as a result of the screening delay.

There are few studies comparable to the one at hand. In the available literature, our study can be compared to a retrospective study conducted in our country, published in 2021, which aimed to assess the impact of the COVID-19 pandemic on DR screening in patients with DM in a healthcare area of Catalonia. They evaluated the period between January 2015 and June 2022, including a total of 16,152 patients, assessing program attendance, metabolic control of DM, and the incidence of DR. The authors observed a reduction in program attendance during the first months of the pandemic, leading to a decrease in DR detection during this time, followed by a recovery in 2021 and 2022. Overall, the authors did not observe an increase in patients with DR related to the COVID-19 pandemic. However, they did note an increase in the number of patients with VTDR during 2021 and 2022, attributing this to the worsening of glycemic control during the pandemic (27). As potential reasons for the discrepancies between our findings and those of Romero-Aroca et al, the sample size in their study is smaller compared to ours and applies only to a single healthcare area in Catalonia, while our study covers the entire Andalusian region (27). Consequently, our study is on a larger scale and evaluates the impact of a complete healthcare system, with a longer follow-up period confirming the recovery of the screening program in the final years of the study. As a limitation, in our case, we do not have data on glycemic control, duration of DM, or antidiabetic treatments. However, we believe that given the long exposure time required for DR development, worsening glycemic control during the pandemic is an unlikely cause for worsening DR outcomes in populations with a high coverage rate such as ours. This is expected in a screening system where the delay time was not extensive. Nevertheless, to assess the global impact of the COVID-19 pandemic on DR, it is necessary to conduct longer-term studies, where the effects of the pandemic on this pathology can be evaluated more comprehensively.

The decision by authorities to interrupt screening programs, such as the APDR, has subsequently been supported in various publications. According to the initial guidelines from the Royal College of Ophthalmologists, the approach to managing DR assessment and follow-up during a pandemic suggested that it may be necessary to postpone DR evaluations in cases of staff shortages, lack of protective equipment, or when infection rates are high (28).

The most significant experience regarding the interruption of screening programs during this health emergency can be found in studies on cancer screening programs. Kregting et al. estimated the impact of a 6-month cessation of breast, colorectal, and cervical cancer screening programs due to the COVID-19 pandemic. They presented five different approaches for restarting the programs and evaluated their estimated impacts on incidence, mortality, and diagnostic capacity (29). In the United States (US), during the first wave of the pandemic, delaying initial lung cancer evaluations was recommended. This resulted in a significant decrease in the incidence of the six most common cancers in the US, including lung cancer (30). This occurred despite the well-established understanding of the implications of delays in diagnosis and treatment for all cancers regarding survival outcomes (31).

### Limitations and strengths

As this is a population-based study with aggregated analysis, we do not have access to individual clinical data such as glycemic control, cardiovascular risk factors, or specific treatments for DM management. However, we believe that the pandemic period alone is insufficient to attribute any worsening of DR in the newly diagnosed cases.

Although the proactive re-engagement of patients following the restart of the APDR has been effective in increasing the number of tests performed, it has not been possible to ensure the systematic inclusion of all patients. This may have introduced a selection bias by excluding the most vulnerable population, where the risk of developing DR could be higher than in the re-engaged cohort.

The follow-up time of our study is relatively short to draw definitive conclusions about the impact of the program’s interruption. It is our intention to extend the follow-up period to conduct a more comprehensive analysis of the situation and assess the full impact that the pandemic may have had on the APDR outcomes in our population.

Given the nature of our dataset—based on aggregated, population-level data without a sampling process—we conducted only descriptive analyses. Although we recognize the potential utility of inferential statistics in many research settings, we consider descriptive analysis appropriate and sufficient to address the objectives and design of this study.

The activity results are consistent with those of other screening programs. The rapid recovery is attributable to proactive re-engagement, which has been essential for restoring the pre-pandemic situation. In any case, long-term follow-up of the cohort is necessary to definitively understand the impact of the pandemic on long-latency complications such as DR, health outcomes in terms of visual disability, and ultimately the quality of life of individuals with DM.

## Conclusions

This study provides the most comprehensive analysis to date of a systematic DR screening program in a large European population. Despite the disruption caused by the COVID-19 pandemic, the APDR demonstrated resilience and a rapid recovery, with screening volumes exceeding pre-pandemic levels by 2022. Importantly, the proportion of VTDR cases remained stable throughout the pandemic period, suggesting that the temporary interruption did not significantly impact VTDR outcomes. These findings underscore the effectiveness of structured, population-based screening systems in mitigating adverse health effects during healthcare crises. However, continued long-term monitoring is essential to assess the full impact on visual outcomes and ensure equitable access, particularly for vulnerable populations who may have experienced delayed or missed screenings.

## Data Availability

The datasets presented in this study can be found in online repositories. The names of the repository/repositories and accession number(s) can be found in the article/supplementary material.
